# Timeframes for “early” mobilisation after abdominal and cardiothoracic surgery: evidence- and consensus-based suggestions of definitions

**DOI:** 10.1186/s12893-026-03729-y

**Published:** 2026-04-18

**Authors:** Monika Fagevik Olsén, Maria Sehlin, Elisabeth Westerdahl, Anna Svensson-Raskh, Linda Block, Cecilia Engström, Malin Nygren-Bonnier, Anna Schandl

**Affiliations:** 1 Department of Neuroscience and Physiology/Health and Rehabilitation, Gothenburg University, Gothenburg, Sweden; 2 Department of Physiotherapy, Sahlgrenska University Hospital, Gothenburg, Sweden; 3 Department of Community Medicine and Rehabilitation, Physiotherapy, Umeå University, Umeå, Sweden; 4 University Health Care Research Centre, Faculty of Medicine, and Health, Örebro University, Örebro, Sweden; 5 Division of Physiotherapy, Department of Neurobiology, Care Sciences and Society, Karolinska Institute, Huddinge, Sweden; 6 Medical Unit Allied Health Professionals, Women’s Health and Allied Health Professionals Theme, Karolinska University Hospital, Stockholm, Sweden; 7 Department of Anaesthesia and Intensive Care, Institute for Clinical Sciences, Sahlgrenska Academy, Gothenburg University, Gothenburg, Sweden; 8 Department of Anaesthesia and Intensive Care, Sahlgrenska University Hospital, Gothenburg, Sweden; 9 Department of Surgery, Institute for Clinical Sciences, Sahlgrenska Academy, Gothenburg University, Gothenburg, Sweden; 10 Department of Perioperative- and Intensive Care, Södersjukhuset, Stockholm, Sweden; 11 Department of Clinical Sciences and Education, Karolinska Institutet, Södersjukhuset, Stockholm, Sweden

**Keywords:** Ambulation, Early mobilisation, Physiotherapy, Postoperative, Rehabilitation, Surgery

## Abstract

**Background and Aim:**

There is currently no consensus on how to define “early” mobilisation following surgery. The aim of this study was to develop definitions of early mobilisation after cardiothoracic and abdominal surgery.

**Methods:**

We conducted a study with multiple methods designs, including a survey, clinical observations and consensus discussions. A national survey investigated Swedish healthcare professionals’ opinions on the typical timeframe for first mobilisation for eight defined categories of surgery, and timeframes for “early” mobilisation. The following consensus discussions were based on the results of the survey and earlier observational data of time to first mobilisation after surgery in 736 patients who had undergone the eight defined surgical procedures.

**Results:**

In total, 503 healthcare professionals completed the survey and 48 healthcare professionals, and 12 patients participated in eight consensus discussions. In the survey, the earliest time range for typical timeframe for first mobilisation (3–6 h) was estimated for patients undergoing cardiac, pulmonary, or minor upper or lower abdominal open surgery and the latest time range (6–9 h) for patients who underwent oesophageal surgery. Across all surgical categories, “early” mobilisation was consistently perceived to occur earlier than the estimated typical timeframe. From the eight consensus discussions, “early” mobilisation was defined as “occurring within 6 hours after surgery, regardless of procedure type”.

**Conclusion:**

In this multiple design national study, “early” mobilisation was defined to be performed within a 6-h time frame after eight categories of abdominal and cardiothoracic surgery. However, the generalisability to other countries is yet to be explored. The findings also highlight the variability in mobilisation times, underscoring the need for tailored preoperative and postoperative care protocols to optimise recovery.

**Trial registration:**

FoU in Sweden” (Research and Development in Sweden) id: 275327 and Clinical Trials NCT04729634 (Registration date: 01/27/2021).

**Supplementary Information:**

The online version contains supplementary material available at 10.1186/s12893-026-03729-y.

## Context and relevance

There is currently no consensus on how to define “early” mobilisation following surgery. In this multiple design national study, “early” mobilisation was defined to be performed within a 6-h time frame after eight categories of abdominal and cardiothoracic surgery. The findings also highlight the variability in mobilisation times, underscoring the need for tailored preoperative and postoperative care protocols to optimise recovery. 

## Introduction

Hospitals worldwide have different interpretations and clinical practices for “early” mobilisation after surgery. Unified international definitions are lacking, complicating clinical practice comparison and improvement. In addition, without a definition, interpreting research findings from studies evaluating postoperative practice, recovery, and complications becomes difficult.

Despite this, guidelines for postoperative care often recommend early mobilisation after surgery [1, 2] but these recommendations are seldom evidence-based and do not often align with the opinions and experiences of healthcare professionals regarding the optimal timing for mobilisation. Early mobilisation typically refers to initiating movement and activity as soon as feasible after surgery. The specific timing for what is considered “early” may vary depending on the type of surgery, the patient’s health condition, and the surgeon’s and healthcare professionals’ recommendations and clinical routines [3–7]. Mobilisation, by definition, includes transition to a more upright position in bed, sitting on the edge of or standing by the bed, sitting in a chair, walking or climbing stairs [8].

Mobilisation has been demonstrated to prevent postoperative pulmonary complications by accelerating and normalising gas exchange, lung volumes, and enhances haemodynamic function [3, 4, 9–11]. Another important benefit is an improvement in well-being and recovery experienced by patients [12]. In addition, enhanced recovery programs after surgery have been implemented at many surgical centres, with mobilisation being one of the components [1, 2]. However, insufficient economic resources and time may hinder the implementation of early mobilisation practices [13–15].

Currently, there is no consensus on the optimal timing for first mobilisation, which may have a negative impact on the effectiveness of patient care [16]. Addressing this gap could enhance surgical and overall outcomes and lead to more standardised and effective postoperative care protocols.

This study aimed to develop definitions of “early” mobilisation after cardiothoracic and abdominal surgery.

## Methods

### Study design

The Survey of MOBilisation after Abdominal and Cardio-Thoracic Surgery (SOMBATA) research project [17] aims to investigate early mobilisation in postoperative patients undergoing abdominal or cardiothoracic surgery in Sweden. In this part, a multiple method design was used, to, in a stepwise process, define early mobilisation after eight defined abdominal or cardiothoracic surgical procedures. The observational part of this study is reported according to the The STrengthening the Reporting of OBservational studies in Epidemiology (STROBE).

The Swedish Ethical Review Authority assessed the project and determined that it did not constitute research requiring ethical approval under the Swedish Ethical Review Act (Reference number 2023-03987-02). Information about the ethics of the study was included in the information about survey and consensus discussion and by contributing, all participants gave their informed consent.

### Healthcare professionals’ perspective on early mobilisation

In the first step of this study, we sought to understand Swedish healthcare professionals’ perspectives on the timing of first postoperative mobilisation. A study-specific survey (Appendix Figure S1) was constructed, pilot-tested for content, and approved by researchers specialised in surgery or anaesthesiology and experienced in mobilisation after abdominal and cardiothoracic surgery.

The survey consisted of 24 questions. The participants were asked to provide their age, sex, profession, current area of work (anaesthesiology/operative ward, intensive/postoperative ward, surgical ward), training status (specialist or not), work experience (total years and years in the specific ward), and hospital type (university, local, or regional).

The remaining questions explored the healthcare professionals’ perspectives on the typical timeframe on first postoperative mobilisation and timeframe for “early” mobilisation after eight different defined surgical categories, all with a duration of anaesthesia exceeding two hours and with inpatient stay:

- cardiac (coronary artery bypass grafting and valve surgery).
- thoracic (pulmonectomy, lobectomy, and pleura).
- oesophageal (incisions including both the thorax, and abdomen).
- major upper abdominal (total gastrectomy, pancreatic total, Whipple´s, and liver procedures).
- minor upper abdominal (cholecystectomy, fundoplication, bariatric procedures, and subtotal gastectomy).
- intestinal (right-sided colectomy and lower anterior resection).
- major lower abdominal (larger oncologic gynaecological, and urological procedures with lymph node resection or cystectomies).
- minor lower abdominal (hysterectomy, oophorectomy, and prostatectomy).

Each surgical category was defined in collaboration with clinical specialists in abdominal and cardiothoracic surgery, with examples of common surgeries within each category. Only the procedures stated were to be considered in each category. The estimations were performed for different surgical techniques (open, minimally invasive, robot-assisted, or hybrid) when applicable. The referenced procedures were elective standard surgeries conducted according to hospital protocols and with no complications.

Mobilisation was defined as moving from bed to at least sitting on the edge of the bed [9]. The participants were instructed to select the time interval that most accurately corresponded to each surgical category (0–3, > 3–6, > 6–9, > 9–12, > 12–15, > 15–18, > 18–21, or > 21–24 h).

The survey was thereafter constructed using the Google web survey, but printed versions were also available. Data was collected from October 2023 to February 2024. Information about the study was disseminated nationwide to healthcare professionals involved in postoperative care and presented at conferences within the field. The survey was subsequently spread to physicians, nurses, assistant nurses, and physiotherapists involved in direct postoperative patient care in hospitals across Sweden. Because the survey was spread via different networks and at conferences it is not known how many healthcare professionals were reached.

### Consensus discussions to define time frames for early mobilisation

In the second step of this study, we invited a convenience sample of Swedish physicians, nurses, assistant nurses, physiotherapists, and patients to participate in consensus discussions to define timeframes for “early” mobilisation for the same eight surgical groups as in the survey and after laparoscopic/thoracoscopic, or robot-assisted laparoscopic/thoracoscopic procedures.

The discussions were designed to include both female and male participants employed at university, regional, or local county hospitals, ensuring representation from all parts of Sweden. The inclusion criterion was clinical practice in the field within the last year. Healthcare representatives were broadly recruited; all hospitals participating in our earlier observational study [17] were contacted and asked to identify possible healthcare professionals, and formal and informal contacts of the research group were used for broadening the inclusion. The patient representatives had undergone surgery in any of the hospitals included in the first SOMBATA study or were found via national patient organisations. We aimed to form each group including at least two representatives of each profession and two patients to capture different perspectives (Table 1). Some of the healthcare representatives participated in more than one discussion. For logistical reasons, the discussions for cardiac and thoracic surgery were conducted together, making these groups larger.

**Table 1:** Number of healthcare professionals and patients participating in the consensus discussions

Surgical category	Physicians	Nurses	Assistant nurses	Physiotherapists	Patients
Cardiac	5	4	1	5	2
Thoracic	5	4	1	5	2
Oesophageal	2	2	1	2	2
Major UAS	2	2	3	3	2
Minor UAS	2	1	0	4	2
Intestinal	2	0	1	3	2
Major LAS	2	1	0	3	2
Minor LAS	2	1	0	3	2

*UAS* upper abdominal surgery, *LAS* lower abdominal surgery

One week before each discussion, all participants received information about how the consensus discussions would be conducted. They were also provided with the results of time until when first mobilisation was performed after the eight defined surgical categories from the first SOMBATA study [17]. This prospective observational cohort study included 1492 participants of which 736 had undergone the surgical categories in focus for the consensus discussions. The data was presented as average time until first mobilisation for each surgical group and technique.

The principal investigator (MFO) participated in all discussions and was assisted by one of the other researchers; one led the discussions towards consensus and the other took field notes.

All discussions were held via the digital platform Zoom. At the start of the discussion all participants presented their roles. Then, the primary investigator reminded of the aim of the discussion and presented the results from the survey and the data from the earlier observational study [17]. The participants were encouraged to give their opinions on the definitions of early mobilisation but also to discuss factors related to patients being mobilised early.

Consistent with the questionnaire study, responses were estimated in the following time frames: 0–3, < 6, <9, < 12, <15, < 18, <21, and < 24 h. However, the intervals were not mandatory. Consensus was reached when at least 70% of participants within the group agreed on the proposed timeframe.

In the field notes the key points were recorded during each discussion. These notes were reviewed and subjected to a basic coding process, paying attention to perspectives on early mobilisation. Key topics were identified through this straightforward thematic sorting. The notes were condensed to highlight insights regarding recommended timeframes for postoperative mobilisation; influencing factors, such as patient condition, preoperative information, and organisational aspects; and areas of common agreement. The results were compiled into a descriptive summary reflecting the main findings and shared viewpoints from the discussions.

### Statistical analysis

Results are presented as descriptive statistics, including means with standard deviations. The Kruskal-Wallis ANOVA test and Pearson’s chi-squared/Fisher´s exact test were employed to analyse differences between professions, with *P* < 0.01 considered significant. IBM SPSS Statistics for Windows, version 28.0 (Armonk, NY: IBM Corp.) was used for the analyses. The work with the questionnaire in this manuscript was prepared following the Consensus-Based Checklist for Reporting of Survey Studies [18].

## Results

The questionnaire was completed by 503 healthcare professionals across hospitals in Sweden. The mean age was 42 ± 12 years, and 81% of respondents were women. Most participants were nurses (*n* = 253) followed by assistant nurses (*n* = 92), physiotherapists (*n* = 80) and physicians (*n* = 78). Of the nurses were 89% specialists (68% anaesthesia/intensive care and 21% surgical care). Of the physicians were 47% anaesthesiologists and 13% surgeon and among the physiotherapists were 5% certified specialists in intensive care and 11% in respiration. The majority of respondents were employed at university hospitals (84%), with others from regional county hospitals (11%) and local county hospitals (5%) (Table 2). The vast majority of participants worked in postoperative units or surgical wards (91%) and had substantial experience in surgical care, with a mean duration of 12 ± 11 years.

**Table 2:** Characteristics of the respondents

		All	Physicians	Nurses	Assistant nurses	Physiotherapists
Total number		503 (100%)	78 (16%)	253 (50%)	92 (18%)	80 (16%)
Age, years		42 (12)	46 (10)	41 (12)	42 (14)	43 (12)
Professional experience, years		16 (12)	19 (11)	15 (11)	17 (15)	15 (10)
Professional experience in surgical care, years		12 (11)	17 (11)	11(10)	10 (12)	11 (10)
Female sex		407 (81%)	33 (42%)	223 (88%)	85 (92%)	66 (83%)
Type of hospital	University	424 (84%)	73 (94%)	223 (88%)	78 (85%)	50 (63%)
	Regional county	56 (11%)	4 (5%)	20 (8%)	11 (12%)	21 (26%)
	Local county	23 (5%)	1 (1%)	10 (4%)	3 (3%)	9 (11%)
Type of ward*	Anaesthesia/operation	52 (10%)	46 (59%)	5 (2%)	1 (1%)	0
	Intensive/postop ward	248 (46%)	32 (41%)	137 (54%)	45 (49%)	34 (43%)
	Surgical ward	245 (45%)	31 (40%)	114 (45%)	46 (50%)	54 (68%)

Data are reported as mean (standard deviation) or n (%). * Combinations possible

### Healthcare professionals’ perspective on early mobilisation

#### Estimation of typical timeframe for first mobilisation after surgery

**Fig. 1:**
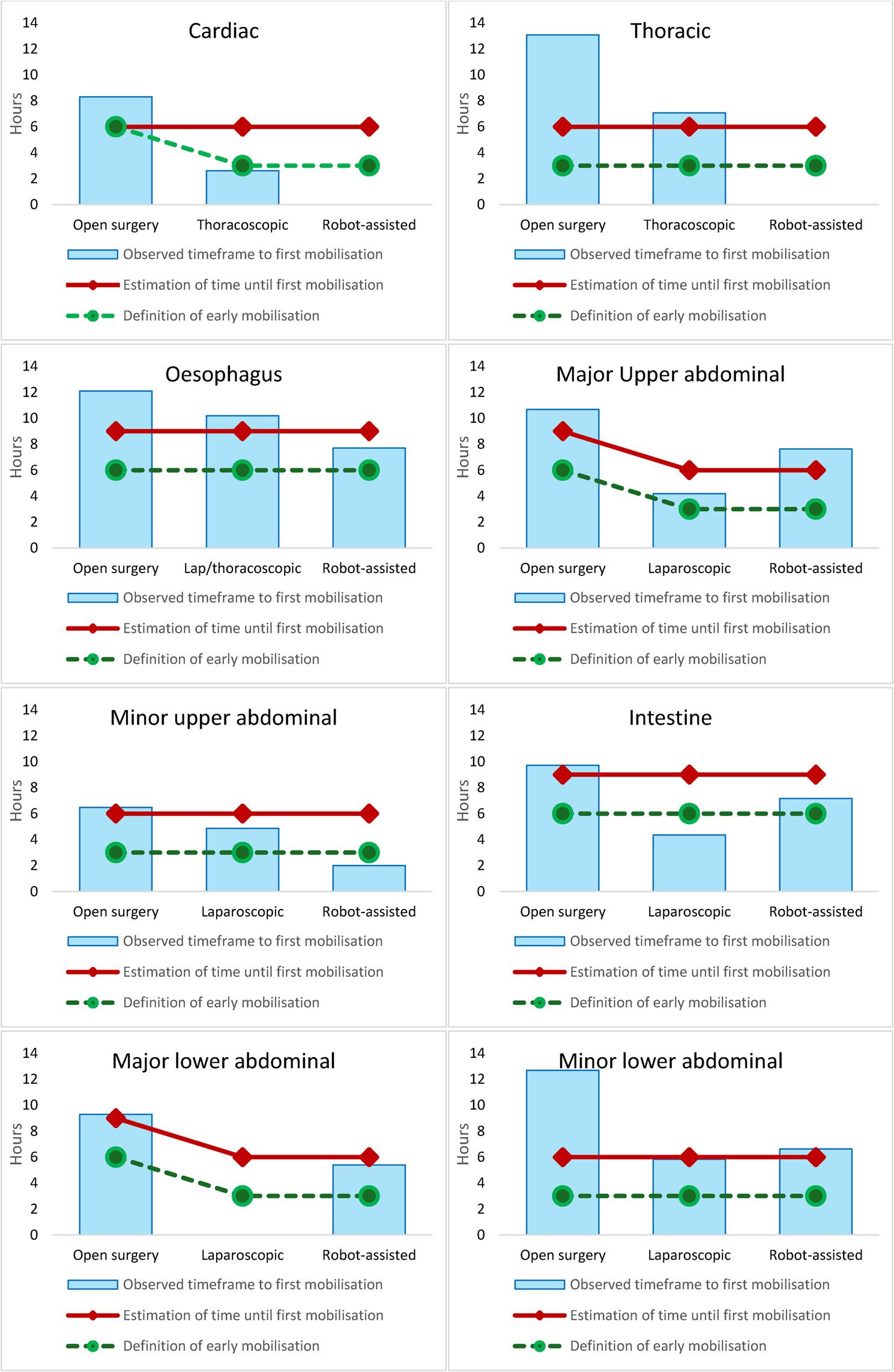
Observed time to and estimated and expected time intervals for first mobilisation after elective surgery

The respondents estimated that the typical timeframe until first mobilisation was within 6 h for patients undergoing cardiac, pulmonary and minor upper and lower abdominal surgery irrespective of surgical technique (Fig. 1). Patients undergoing oesophageal or intestinal surgery, irrespective of surgical technique were estimated to be mobilised within 9 h. After major upper and lower abdominal surgery, the respondents estimated mobilisation to be within 9 h after open surgery and 6 h after the minimally invasive procedures.

Healthcare professionals’ estimations across various professions were generally consistent across different surgery types, with few exceptions. Physicians estimated a later time interval to mobilisation for patients who underwent major lower abdominal open surgery (9–12 h) compared to other professions (6–9 h, *p* = 0.005). Similarly, for minimally invasive surgery, physicians’ estimations were later (6–9 h) for major lower abdominal surgery compared to other professions (3–6 h, *p* = 0.002). Physiotherapists estimated a later time interval for mobilisation in patients with minor lower abdominal open surgery compared to other professions (6–9 h versus 3–6 h, *p* = 0.009). Moreover, assistant nurses and physiotherapists estimated a later time interval to mobilisation for minimally invasive cardiac surgery and minor lower abdominal surgery than other professions (Supplementary Table S1).

#### Estimated timeframe for “early” mobilisation

For all types of surgeries, early mobilisation was expected to be one time interval earlier than the estimated initial mobilisation (Fig. 1). Cardiac surgery was an exception, showing better conformity in the timing of first mobilisation. No significant differences in the reported time intervals for early mobilisation were observed across different professions (data not shown). 

### Consensus discussions to define time frames for “early” mobilisation

**Fig. 2:**
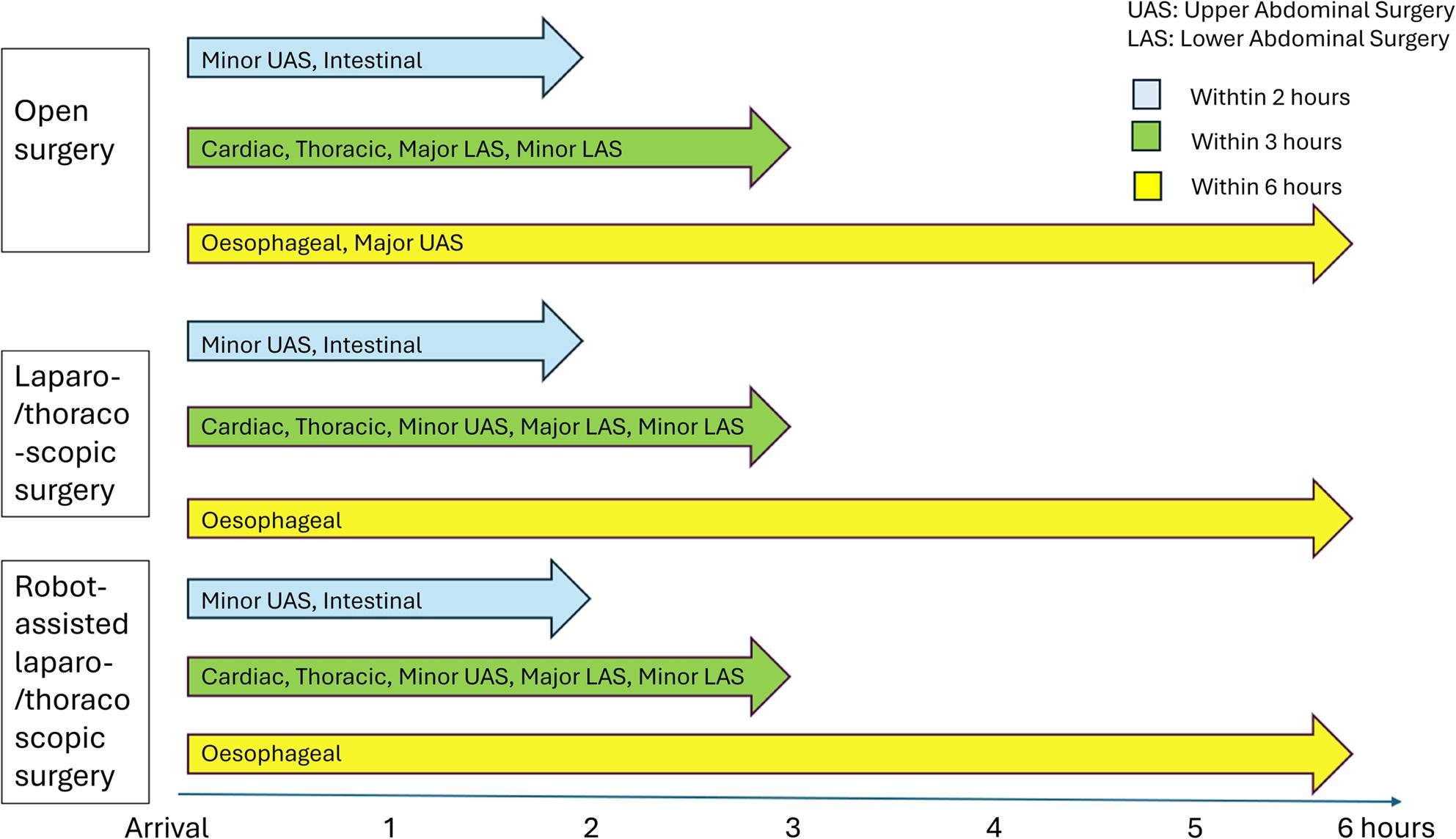
Definitions of “early” mobilisation

Consensus was reached in all groups regarding the time frames for the eight specific surgical groups (Fig. 2). In summary, all consensus groups agreed upon that mobilisation could be recommended to start within 0–6 h after surgery, with specific timelines depending on the type of surgery and the patient’s condition. All participants agreed on the importance of early mobilisation and that it is feasible with the right conditions and support.

The field notes were summarised into the following topics:

#### Mobilisation timeframes

Mobilisation could be recommended to occur within 0–6 h after surgery depending on the type of surgery and the patient’s stability. For minor procedures, mobilisation was recommended within 2 h, whereas major procedures allowed for mobilisation within 3–6 h. In the consensus groups on cardiothoracic and oesophageal surgery, mobilisation was, in some cases, related to the removal of pericardial or pleural drainage.

#### Patient condition

Mobilisation should occur when the patient is awake, stable regarding respiratory and circulatory function and pain adequately managed. The patient’s wellbeing and stability are crucial factors.

#### Preoperative information

Effective preoperative information and multidisciplinary care are essential for successful mobilisation. A well-informed and motivated patient can be mobilised more easily.

#### Role of healthcare personnel

Engaged healthcare personnel who actively guide patients during mobilisation are important. The experience of the personnel is crucial to mitigating risks associated with mobilisation. Mobilisation is facilitated by the presence of physiotherapists and other trained and educated healthcare staff.

#### Organisational factors

Mobilisation can be influenced by staffing levels, the time of day the surgery is completed, and resource availability. It is important to have structures and routines in place to ensure timely mobilisation.

#### Patient experience

Being connected to tubes and wires can be intimidating and uncomfortable for the patient. Good communication and support from the staff are important to reduce anxiety and fear.

## Discussion

In this study, “early” mobilisation for eight categories of surgery has been investigated and defined. The study is based on healthcare professionals´ estimations of typical timeframes for first mobilisation and when it should occur to be considered “early”, and consensus discussions between healthcare professionals and patients. In addition, the discussions were based on the participating people´s own experiences, the results of the survey and but also the observed exact time for first mobilisation from the first SOMBATA study [17]. According to these discussions, the intervention should be performed within 6 h postoperatively to be defined as “early”.

Previous studies and guidelines recommend postoperative mobilisation (bedside sitting, standing, or walking short distances) within 24 to 48 h of surgery [3, 4, 5, 7, 8]. However, whether this timeframe is considered “early” is debatable. In the first part of the SOMBATA project, 52% of the patients were mobilised to at least sitting on the edge of the bed within 6 h after surgery [17]. In two other trials from our research group, it was found that mobilisation can be initiated within the first hours after abdominal surgery [3, 4]. In addition, in an interview study of 23 patients mobilised out of bed within 2 h of major abdominal surgery, it was found that mobilisation was well received provided that competent support from healthcare staff was available [12]. Given these findings, along with the results from the current study, early mobilisation within the first 6 h after surgery appears feasible, but other trials are needed to determine the optimal timing for safe mobilisation.

In this study, the healthcare personnel estimated that patients usually are mobilised within a timeframe of 9 h after any type of surgery. When the results were compared to the observed actual time to initial mobilisation after surgery [17], it was found that mobilisation occurred later than expected for five of the surgical categories, Fig. 1. This indicates that healthcare professionals tend to believe mobilisation happens earlier than it does. The reason for this discrepancy remains unknown and may have different reasons for healthcare professions. Surgeons in Sweden, who prescribe early mobilisation based on perioperative status, may estimate times differently as they are often not involved in the actual postoperative mobilisation. Swedish physiotherapists work most often only during office hours. Understanding others’ expertise in the context of interprofessional collaboration and recognising overlapping focus is crucial to minimising differences in perspective. The discrepancy is essential knowledge for accurately planning the intensive and resource-demanding postoperative care.

However, successful mobilisation heavily relies on adequate resources and competent staff [13–15, 19, 20]. Different professions may take on varied responsibilities for initiating early mobilisation [21]. Therefore, it is crucial to have competent staff available for early mobilisation in the postoperative phase, especially for patients with comorbidities and those with an increased risk of postoperative complications. Another important aspect to consider that was raised during the consensus discussions is whether there is a risk of performing mobilisation too early. Few trials have investigated the optimal timing for safe mobilisation regarding adverse events, such as falls, syncope, or arrhythmia. Nevertheless, the importance of monitoring patients during early mobilisation must be emphasised.

### Study strengths and limitations

This national multiple method study provides a comprehensive exploration of how “early” mobilisation is perceived and can be defined following cardiothoracic and abdominal surgery. By integrating survey responses from over 500 healthcare professionals with consensus discussions involving both clinicians and patients, the study offers a robust and inclusive definition.

One of the key strengths of this study is its broad national scope and large sample size, which enhances the representativeness of the findings within the Swedish healthcare context. The different designs allowed for triangulation of quantitative and qualitative data, strengthening the validity of the conclusions. Importantly, the inclusion of both healthcare professionals and patients ensured that the final definition reflects both clinical feasibility and patient-centred perspectives.

However, some limitations should be acknowledged. First, the study’s findings may not be directly generalisable to other countries, where healthcare systems, staffing models, and postoperative care practices may differ. Second, the reliance on estimated timeframes and professional opinions introduces the potential for response bias and may not accurately reflect actual clinical practice. Third, the response rate of the survey is not known as the survey was spread via networks and at conference. Additionally, while the definition of “early” mobilisation as occurring within six hours provides a useful benchmark, it may oversimplify the complex clinical realities faced by individual patients, such as comorbidities or postoperative complications. Finally, consensus discussions, while valuable, may be influenced by group dynamics, potentially limiting the diversity of viewpoints represented in the final definition.

Despite these limitations, the study contributes significantly to the ongoing dialogue around postoperative care and recovery. It provides a clear, actionable definition of “early” mobilisation that can inform clinical guidelines, quality improvement initiatives, and future research. Further studies are needed to validate this definition in international contexts and to explore its impact on patient outcomes.

## Conclusions

This national, multiple methods study of perioperative healthcare professionals in Sweden found a high degree of consensus to define ‘early’ postoperative mobilization as that occurring within the first 6 h of the surgery. However, the generalisability to other countries is yet to be explored. The findings also highlight the variability in mobilisation times based on the type and method of surgery, underscoring the need for tailored preoperative and postoperative care protocols to individual patient and procedural needs, rather than relying on a one-size-fits-all approach. 

## Supplementary Information

Supplementary Material 1.

Supplementary Material 2.

## Data Availability

Data will be shared by first author on reasonable request.

## Abbreviation

SOMBATA: The Survey of MOBilisation after Abdominal and Cardio-Thoracic Surgery

## References

[1] Gustafsson UO, Scott MJ, Hubner M, Nygren J, Demartines N, Francis N, et al. Guidelines for Perioperative Care in Elective Colorectal Surgery: Enhanced Recovery After Surgery (ERAS((R)) Society Recommendations: 2018. World J Surg. 2019;43:659–95. 30426190 10.1007/s00268-018-4844-y

[2] Lavu H, McCall NS, Winter JM, Burkhart RA, Pucci M, Leiby BE, et al. Enhancing patient outcomes while containing costs after complex abdominal operation: a randomized controlled trial of the whipple accelerated recovery pathway. J Am Coll Surg. 2019;228:415–24. 30660818 10.1016/j.jamcollsurg.2018.12.032 PMC8158656

[3] Fagevik Olsen M, Becovic S, Dean E. Short-term effects of mobilization on oxygenation in patients after open surgery for pancreatic cancer: a randomized controlled trial. BMC Surg. 2021;21:185. 33827537 10.1186/s12893-021-01187-2 PMC8028068

[4] Svensson-Raskh A, Schandl AR, Stahle A, Nygren-Bonnier M, Fagevik Olsén M. Mobilization started within 2 hours after abdominal surgery improves peripheral and arterial oxygenation: a single-center randomized controlled trial. Phys Ther. 2021;101:pzab094. 33742678 10.1093/ptj/pzab094 PMC8136304

[5] Souza Possa S, Braga Amador C, Meira Costa A, Takahama Sakamoto E, Seiko Kondo Cm Maida, Vasconcellos AL, et al. Implementation of a guideline for physical therapy in the postoperative period of upper abdominal surgery reduces the incidence of atelectasis and length of hospital stay. Rev Port Pneumol. 2014;20:69–77. 24290563 10.1016/j.rppneu.2013.07.005

[6] de Almeida EPM, de Almeida JP, Landoni G, Galas FRBG, Fukushima JT, Fominskiy E, et al. Early mobilization programme improves functional capacity after major abdominal cancer surgery: a randomized controlled trial. Br J Anaesth. 2017;119:900–7. 28981596 10.1093/bja/aex250

[7] Hashem MD, Nelliot A, Needham DM. Early mobilization and rehabilitation in the ICU: moving back to the future. Respir Care. 2016;61:971–9. 27094396 10.4187/respcare.04741

[8] Tazreean R, Nelson G, Twomey R. Early mobilization in enhanced recovery after surgery pathways: current evidence and recent advancements. J Comp Eff Res. 2022;11:121–9. 35045757 10.2217/cer-2021-0258

[9] Kanejima Y, Shimogai T, Kitamura M, Ishihara K, Izawa KP. Effect of early mobilization on physical function in patients after cardiac surgery: a systematic review and meta-analysis. Int J Environ Res Public Health. 2020;17:7091. 10.3390/ijerph17197091 PMC7578990 32998202

[10] Fjerbaek A, Westerdahl E, Andreasen J, Thomsen LP, Brocki BC. Change of position from a supine to a sitting position increases pulmonary function early after cardiac surgery. Eur J Physiother. 2019;22:313–7.

[11] Waxman K. Hemodynamic and metabolic changes during and following operation. Crit Care Clin. 1987;3:241–50. 3332198

[12] Svensson-Raskh A, Schandl A, Holdar U, Fagevik Olsén M, Nygren-Bonnier M. I Have everything to win and nothing to lose: patient experiences of mobilization out of bed immediately after abdominal surgery. Phys Ther. 2020;100:2079–89. 32941610 10.1093/ptj/pzaa168 PMC7720638

[13] Sepulveda-Pacsi AL, Soderman M, Kertesz L. Nurses’ perceptions of their knowledge and barriers to ambulating hospitalized patients in acute settings. Appl Nurs Res. 2016;32:117–21. 27969013 10.1016/j.apnr.2016.06.001

[14] Svensson-Raskh A, Olsen MF, Nygren-Bonnier M, Schandl A. Healthcare professionals’ experiences of mobilising adult patients out of bed shortly after major abdominal surgery - a qualitative study. Eur J Physiother. 2024;26:273–9.

[15] Alawadi ZM, Leal I, Phatak UR, Flores-Gonzales JR, Holihan JL, Karanjawala BE, et al. Facilitators and barriers of implementing enhanced recovery in colorectal surgery at a safety net hospital: A provider and patient perspective. Surgery. 2016;159:700–12. 26435444 10.1016/j.surg.2015.08.025

[16] Castelino T, Fiore JF Jr., Niculiseanu P, Landry T, Augustin B, Feldman LS. The effect of early mobilization protocols on postoperative outcomes following abdominal and thoracic surgery: A systematic review. Surgery. 2016;159:991–1003. 26804821 10.1016/j.surg.2015.11.029

[17] Fagevik Olsen M, Sehlin M, Westerdahl E, Schandl A, Block L, Nygren-Bonnier M, Svensson-Raskh A. First mobilisation after abdominal and cardiothoracic surgery: when is it actually performed? A national, multicentre, cross-sectional study. BMJ Open. 2024;14:e082239. 38423778 10.1136/bmjopen-2023-082239 PMC10910679

[18] Sharma A, Minh Duc NT, Luu Lam Thang T, Hai Nam N, Ng SJ, Abbas KS, et al. A Consensus-Based Checklist for Reporting of Survey Studies (CROSS). J Gen Intern Med. 2021;36:3179–87. 33886027 10.1007/s11606-021-06737-1 PMC8481359

[19] Lyon A, Solomon MJ, Harrison JD. A qualitative study assessing the barriers to implementation of enhanced recovery after surgery. World J Surg. 2014;38:1374–80. 24385194 10.1007/s00268-013-2441-7

[20] Brown CJ, Williams BR, Woodby LL, Davies LL, Allman RM. Barriers to mobility during hospitalization from the perspectives of older patients and their nurses and physicians. J Hosp Med. 2007;2:305–13. 17935241 10.1002/jhm.209

[21] Garzon-Serrano J, Ryan C, Waak K, Tully S, Bittner EA, Chipman DW, et al. Early mobilization in critically ill patients: patients’ mobilization level depends on health care provider’s profession. PM R. 2011;3:307–13. 21497316 10.1016/j.pmrj.2010.12.022

