# Supplementary material for: Timeframes for “early” mobilisation after abdominal and cardiothoracic surgery: evidence- and consensus-based suggestions of definitions
Source: BMC Surg. 2026 Apr 18;26:281. doi: 10.1186/s12893-026-03729-y (PMC13091274; doi:10.1186/s12893-026-03729-y)
Supplement: Supplementary file 1 — Supplementary Material 1. [file 12893_2026_3729_MOESM1_ESM.docx]

Supplementary Table S1. Estimations of typical timeframe during which patients usually are mobilised after surgery.

|  |  | **Physicians**  n=78 | **Nurses** n=253 | **Assistant nurses**  n=92 | **Physiotherapists**  n=80 |  |
| --- | --- | --- | --- | --- | --- | --- |
|  |  | Median time interval  in hours (IQR) | Median time interval  in hours (IQR) | Median time interval  in hours (IQR) | Median time interval  in hours (IQR) | P-value |
| **Open surgery** | Cardiac | >3-6 (0-3; >3-6) | >3-6 (0-3; >3-6) | >3-6 (0-3; >3-6) | >3-6 (>3-6; >9-12) | 0.044 |
|  | Pulmonary | 0-3 (0-3; >3-6) | 0-3 (0-3; >3-6) | 0-3 (0-3; >3-6) | >3-6 (0-3; >3-6) | 0.253 |
|  | Oesophageal | >3-6 (<3-6; >3-6) | >3-6 (<3-6; >6-9) | >3-6 (<3-6; >6-9) | >3-6 (0-3; >3-6) | 0.315 |
|  | Major upper abdominal | >3-6 (0-3; >3-6) | >3-6 (0-3; >3-6) | >3-6 (0-3; >6-9) | >3-6 (0-3; >3-6) | 0.346 |
|  | Minor upper abdominal | 0-3 (0-3; >3-6) | 0-3 (0-3; >3-6) | 0-3 (0-3; >3-6) | 0-3 (0-3; >3-6) | 0.903 |
|  | Colon | >3-6 (0-3; >3-6) | >3-6 (0-3; >3-6) | >3-6 (0-3; >3-6) | >3-6 (0-3; >3-6) | 0.378 |
|  | Major lower abdominal | >3-6 (0-3; >6-9) | >3-6 (0-3; >6-9) | >3-6 (0-3; >6-9) | 0-3 (0-3; >3-6) | 0.115 |
|  | Minor lower abdominal | 0-3 (0-3; >3-6) | 0-3 (0-3; >3-6) | 0-3 (0-3; >3-6) | 0-3 (0-3; >3-6) | 0.357 |
| **Minimally invasive**  **surgery** | Cardiac | 0-3 (0-3; 0-3) | 0-3 (0-3; >3-6) | >3-6 (0-3; >3-6) | >3-6 (0-3; >3-6) | 0.147 |
|  | Pulmonary | 0-3 (0-3; 0-3) | 0-3 (0-3; >3-6) | >3-6 (0-3; >3-6) | 0-3 (0-3; >3-6) | 0.332 |
|  | Oesophageal | >3-6 (0-3; >3-6) | >3-6 (0-3; >3-6) | >3-6 (0-3; >6-9) | >3-6 (0-3; >3-6) | 0.244 |
|  | Major upper abdominal | 0-3 (0-3; >3-6) | 0-3 (0-3; >3-6) | 0-3 (0-3; >3-6) | 0-3 (0-3; >3-6) | 0.477 |
|  | Minor upper abdominal | 0-3 (0-3; 0-3) | 0-3 (0-3; 0-3) | 0-3 (0-3>3-6) | 0-3 (0-3; >3-6) | 0.326 |
|  | Colon | 0-3 (0-3; >3-6) | 0-3 (0-3; >3-6) | 0-3 (0-3; >3-6) | 0-3 (0-3; >3-6) | 0.223 |
|  | Major lower abdominal | 0-3 (0-3; >3-6) | 0-3 (0-3; >3-6) | 0-3 (0-3; >3-6) | 0-3 (0-3; >3-6) | 0.511 |
|  | Minor lower abdominal | 0-3 (0-3; 0-3) | 0-3 (0-3; 0-3) | 0-3 (0-3; >3-6) | 0-3 (0-3; 0-3) | 0.104 |
| **Minimally invasive or robot-assisted**  **surgery** | Cardiac | 0-3 (0-3; 0-3) | 0-3 (0-3; >3-6) | >3-6 (0-3; >3-6) | >3-6 (0-3; >3-6) | 0.114 |
|  | Pulmonary | 0-3 (0-3; >3-6) | 0-3 (0-3; >3-6) | 0-3 (0-3; >3-6) | 0-3 (0-3; >3-6) | 0.984 |
|  | Major upper abdominal | 0-3 (0-3; >3-6) | 0-3 (0-3; >3-6) | >3-6 (0-3; >3-6) | 0-3 (0-3; >3-6) | 0.773 |
|  | Minor upper abdominal | 0-3 (0-3; 0-3) | 0-3 (0-3; 0-3) | 0-3 (0-3; >3-6) | 0-3 (0-3; >3-6) | 0.560 |
|  | Colon | 0-3 (0-3; >3-6) | 0-3 (0-3; >3-6) | 0-3 (0-3; >3-6) | 0-3 (0-3; >3-6) | 0.406 |
|  | Major lower abdominal | 0-3 (0-3; >3-6) | 0-3 (0-3; >3-6) | 0-3 (0-3; >3-6) | 0-3 (0-3; >3-6) | 0.310 |
|  | Minor lower abdominal | 0-3 (0-3; 0-3) | 0-3 (0-3; >3-6) | 0-3 (0-3; >3-6) | 0-3 (0-3; >3-6) | 0.342 |
| **Hybrid**  **surgery** | Oesophageal | >3-6 (0-3; >6-9) | >3-6 (>3-6; >6-9) | >3-6 (>3-6; >6-9) | >3-6 (0-3; >3-6) | 0.362 |

P<0.01 refers to significant differences between groups. IQR=interquartile range,
