# Supplementary material for: Timeframes for “early” mobilisation after abdominal and cardiothoracic surgery: evidence- and consensus-based suggestions of definitions
Source: BMC Surg. 2026 Apr 18;26:281. doi: 10.1186/s12893-026-03729-y (PMC13091274; doi:10.1186/s12893-026-03729-y)
Supplement: Supplementary file 2 — Supplementary Material 2. [file 12893_2026_3729_MOESM2_ESM.docx]

### Survey of MOBilisation after abdominal and cardiothoracic surgery: SOMBATA 2.0

Mobilisation after abdominal and cardiothoracic surgery


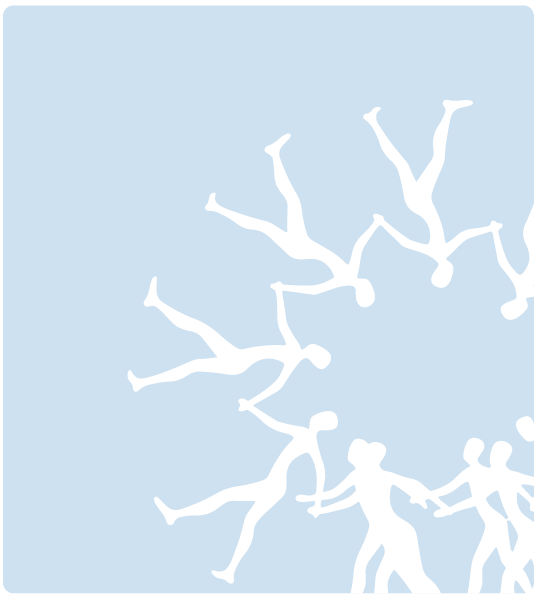


Mobilisation after abdominal and cardiothoracic surgery
Early mobilisation following abdominal and cardiothoracic surgery is not clearly defined in the scientific literature. The aim of this survey is to explore your perception of what constitutes “early” mobilisation. The results of this survey will contribute to the ongoing work of defining early mobilisation within Swedish healthcare.

Completion of the survey implies consent to participate. Participation in the study involves no specific risks or consequences. The benefit of participating is that the study will generate increased knowledge regarding postoperative care.

**Background Information**

This section collects background information about you as a respondent.

**1. Professional Category**
☐ Physician
☐ Registered Nurse
☐ Assistant Nurse
☐ Physiotherapist

**2. Area of Work** *(Please tick the relevant area)*
☐ Anaesthesia/Surgery
☐ ICU/Postoperative Ward
☐ Surgical Ward

**3. Gender**
☐ Female
☐ Male

**4. Age:** ______ years

**5. Years in Profession:** ______ years

**6. Years in Surgery/Anaesthesia/Intensive Care/Postoperative Care:** ______ years

**7. Specialist Training**
☐ No
☐ Yes, in: ______________________

**8. What type of hospital do you work at?**
☐ University Hospital
☐ County Hospital
☐ District Hospital

You will now be asked 8 questions which you are requested to estimate:

**a)** when patients undergoing standardised and defined planned surgical procedures with a normal postoperative course are *usually* mobilised after surgery
**b)** what you consider to be *early* mobilisation for each patient category

For procedures performed using different techniques (open or laparo-/thoracoscopic, with or without robotic assistance), please indicate the timing for each technique.
If no procedures are performed using a specific technique, leave that column blank.
If you have no experience with a particular patient category, skip the question.

**Mobilisation** is defined as a change in position where the patient is at minimum sitting on the edge of the bed.

**Patient Categories:**

1. Cardiac surgery
2. Pulmonary surgery
3. Thoracoabdominal surgery
4. Major upper abdominal surgery
5. Minor upper abdominal surgery
6. Bowel resection
7. Major lower abdominal surgery
8. Minor lower abdominal surgery

**Cardiac Surgery**

Have you worked in the past year with patients who have undergone cardiac surgery?
*(Procedures such as coronary artery bypass grafting [CABG] and/or valve surgery [aortic/mitral])*

☐ No, proceed to the next question section.
☐ Yes, tick the time intervals you believe best match each category.

|  | **How many hours after the end of surgery do you estimate that patients are usually mobilised?** | | | **How many hours after the end of surgery do you consider mobilisation should occur for it to be considered early?** | | |
| --- | --- | --- | --- | --- | --- | --- |
| **Within number of hours** | **Open** | **Thoracoscopic** | **Robot-assisted thoracoscopic** | **Open** | **Thoracoscopic** | **Robot-assisted thoracoscopic** |
| 0–3 | ☐ | ☐ | ☐ | ☐ | ☐ | ☐ |
| >3–6 | ☐ | ☐ | ☐ | ☐ | ☐ | ☐ |
| >6–9 | ☐ | ☐ | ☐ | ☐ | ☐ | ☐ |
| >9–12 | ☐ | ☐ | ☐ | ☐ | ☐ | ☐ |
| >12–15 | ☐ | ☐ | ☐ | ☐ | ☐ | ☐ |
| >15–18 | ☐ | ☐ | ☐ | ☐ | ☐ | ☐ |
| >18–21 | ☐ | ☐ | ☐ | ☐ | ☐ | ☐ |
| >21–24 | ☐ | ☐ | ☐ | ☐ | ☐ | ☐ |

**Thoracic Surgery**

Have you worked in the past year with patients who have undergone pulmonary surgery?
*(Procedures involving the lungs, pleura, or diaphragm, often performed due to cancer. The following procedures are excluded: pneumothorax, infections, traumatic injuries, and chest wall malformations)*

☐ No, proceed to the next question section.
☐ Yes, tick the time intervals you believe best match each category.

|  | **How many hours after the end of surgery do you estimate that patients are usually mobilised?** | | | **How many hours after the end of surgery do you consider mobilisation should occur for it to be considered early?** | | |
| --- | --- | --- | --- | --- | --- | --- |
| **Within number of hours** | **Open** | **Thoracoscopic** | **Robot-assisted thoracoscopic** | **Open** | **Thoracoscopic** | **Robot-assisted thoracoscopic** |
| 0–3 | ☐ | ☐ | ☐ | ☐ | ☐ | ☐ |
| >3–6 | ☐ | ☐ | ☐ | ☐ | ☐ | ☐ |
| >6–9 | ☐ | ☐ | ☐ | ☐ | ☐ | ☐ |
| >9–12 | ☐ | ☐ | ☐ | ☐ | ☐ | ☐ |
| >12–15 | ☐ | ☐ | ☐ | ☐ | ☐ | ☐ |
| >15–18 | ☐ | ☐ | ☐ | ☐ | ☐ | ☐ |
| >18–21 | ☐ | ☐ | ☐ | ☐ | ☐ | ☐ |
| >21–24 | ☐ | ☐ | ☐ | ☐ | ☐ | ☐ |

**Oesophageal Surgery**

Have you worked in the past year with patients who have undergone oesophageal resection involving thoracoabdominal surgery?

☐ No, proceed to the next question section.
☐ Yes, tick the time intervals you believe best match each category.

|  | **How many hours after the end of surgery do you estimate that patients are usually mobilised?** | | | **How many hours after the end of surgery do you consider mobilisation should occur for it to be considered early?** | | |
| --- | --- | --- | --- | --- | --- | --- |
| **Within number of hours** | **Open** | **Thorac- laparoscopic** | **Robot-assisted thoraco-/laparoscopic** | **Open** | **Thoraco-laparoscopic** | **Robot-assisted thoraco-/laparoscopic** |
| 0–3 | ☐ | ☐ | ☐ | ☐ | ☐ | ☐ |
| >3–6 | ☐ | ☐ | ☐ | ☐ | ☐ | ☐ |
| >6–9 | ☐ | ☐ | ☐ | ☐ | ☐ | ☐ |
| >9–12 | ☐ | ☐ | ☐ | ☐ | ☐ | ☐ |
| >12–15 | ☐ | ☐ | ☐ | ☐ | ☐ | ☐ |
| >15–18 | ☐ | ☐ | ☐ | ☐ | ☐ | ☐ |
| >18–21 | ☐ | ☐ | ☐ | ☐ | ☐ | ☐ |
| >21–24 | ☐ | ☐ | ☐ | ☐ | ☐ | ☐ |

**Major Upper Abdominal Surgery**

**Major Upper Abdominal Surgery**
Have you worked in the past year with patients who have undergone major upper abdominal surgery?
*(Procedures such as gastrectomy, total pancreatic resection, Whipple’s procedure, and liver resection)*

☐ No, proceed to the next question section.
☐ Yes, tick the time intervals you believe best match each category.

|  | **How many hours after the end of surgery do you estimate that patients are usually mobilised?** | | | **How many hours after the end of surgery do you consider mobilisation should occur for it to be considered early?** | | |
| --- | --- | --- | --- | --- | --- | --- |
| **Within number of hours** | **Open** | **Laparoscopic** | **Robot-assisted laparoscopic** | **Open** | **Laparoscopic** | **Robot-assisted laparoscopic** |
| 0–3 | ☐ | ☐ | ☐ | ☐ | ☐ | ☐ |
| >3–6 | ☐ | ☐ | ☐ | ☐ | ☐ | ☐ |
| >6–9 | ☐ | ☐ | ☐ | ☐ | ☐ | ☐ |
| >9–12 | ☐ | ☐ | ☐ | ☐ | ☐ | ☐ |
| >12–15 | ☐ | ☐ | ☐ | ☐ | ☐ | ☐ |
| >15–18 | ☐ | ☐ | ☐ | ☐ | ☐ | ☐ |
| >18–21 | ☐ | ☐ | ☐ | ☐ | ☐ | ☐ |
| >21–24 | ☐ | ☐ | ☐ | ☐ | ☐ | ☐ |

**Minor Upper Abdominal Surgery**

**Minor Upper Abdominal Surgery**
Have you worked in the past year with patients who have undergone minor upper abdominal surgery?
*(Procedures such as partial gastrectomy, cholecystectomy, fundoplication, and bariatric surgery)*

☐ No, proceed to the next question section.
☐ Yes, tick the time intervals you believe best match each category.

|  | **How many hours after the end of surgery do you estimate that patients are usually mobilised?** | | | **How many hours after the end of surgery do you consider mobilisation should occur for it to be considered early?** | | |
| --- | --- | --- | --- | --- | --- | --- |
| **Within number of hours** | **Open** | **Laparoscopic** | **Robot-assisted laparoscopic** | **Open** | **Laparoscopic** | **Robot-assisted laparoscopic** |
| 0–3 | ☐ | ☐ | ☐ | ☐ | ☐ | ☐ |
| >3–6 | ☐ | ☐ | ☐ | ☐ | ☐ | ☐ |
| >6–9 | ☐ | ☐ | ☐ | ☐ | ☐ | ☐ |
| >9–12 | ☐ | ☐ | ☐ | ☐ | ☐ | ☐ |
| >12–15 | ☐ | ☐ | ☐ | ☐ | ☐ | ☐ |
| >15–18 | ☐ | ☐ | ☐ | ☐ | ☐ | ☐ |
| >18–21 | ☐ | ☐ | ☐ | ☐ | ☐ | ☐ |
| >21–24 | ☐ | ☐ | ☐ | ☐ | ☐ | ☐ |

**Intestinal Resection**

**Bowel Resection**
Have you worked in the past year with patients who have undergone intestinal resection?
*(Procedures such as right-sided colectomy or low anterior resection)*

☐ No, proceed to the next question section.
☐ Yes, tick the time intervals you believe best match each category.

|  | **How many hours after the end of surgery do you estimate that patients are usually mobilised?** | | | **How many hours after the end of surgery do you consider mobilisation should occur for it to be considered early?** | | |
| --- | --- | --- | --- | --- | --- | --- |
| **Within number of hours** | **Open** | **Laparoscopic** | **Robot-assisted laparoscopic** | **Open** | **Laparoscopic** | **Robot-assisted laparoscopic** |
| 0–3 | ☐ | ☐ | ☐ | ☐ | ☐ | ☐ |
| >3–6 | ☐ | ☐ | ☐ | ☐ | ☐ | ☐ |
| >6–9 | ☐ | ☐ | ☐ | ☐ | ☐ | ☐ |
| >9–12 | ☐ | ☐ | ☐ | ☐ | ☐ | ☐ |
| >12–15 | ☐ | ☐ | ☐ | ☐ | ☐ | ☐ |
| >15–18 | ☐ | ☐ | ☐ | ☐ | ☐ | ☐ |
| >18–21 | ☐ | ☐ | ☐ | ☐ | ☐ | ☐ |
| >21–24 | ☐ | ☐ | ☐ | ☐ | ☐ | ☐ |

**Major Lower Abdominal Surgery**

**Major Lower Abdominal Surgery**
Have you worked in the past year with patients who have undergone major lower abdominal surgery?
*(Procedures such as extensive gynecological or urological surgery (including lymph node dissection) or cystectomy)*

☐ No, proceed to the next question section.
☐ Yes, tick the time intervals you believe best match each category.

|  | **How many hours after the end of surgery do you estimate that patients are usually mobilised?** | | | **How many hours after the end of surgery do you consider mobilisation should occur for it to be considered early?** | | |
| --- | --- | --- | --- | --- | --- | --- |
| **Within number of hours** | **Open** | **Laparoscopic** | **Robot-assisted laparoscopic** | **Open** | **Laparoscopic** | **Robot-assisted laparoscopic** |
| 0–3 | ☐ | ☐ | ☐ | ☐ | ☐ | ☐ |
| >3–6 | ☐ | ☐ | ☐ | ☐ | ☐ | ☐ |
| >6–9 | ☐ | ☐ | ☐ | ☐ | ☐ | ☐ |
| >9–12 | ☐ | ☐ | ☐ | ☐ | ☐ | ☐ |
| >12–15 | ☐ | ☐ | ☐ | ☐ | ☐ | ☐ |
| >15–18 | ☐ | ☐ | ☐ | ☐ | ☐ | ☐ |
| >18–21 | ☐ | ☐ | ☐ | ☐ | ☐ | ☐ |
| >21–24 | ☐ | ☐ | ☐ | ☐ | ☐ | ☐ |

**Minor Lower Abdominal Surgery**

**Minor Lower Abdominal Surgery**
Have you worked in the past year with patients who have undergone minor lower abdominal surgery?
*(Procedures such as hysterectomy, prostatectomy, and ovarian surgery)*

☐ No, proceed to the next question section.
☐ Yes, tick the time intervals you believe best match each category.

|  | **How many hours after the end of surgery do you estimate that patients are usually mobilised?** | | | **How many hours after the end of surgery do you consider mobilisation should occur for it to be considered early?** | | |
| --- | --- | --- | --- | --- | --- | --- |
| **Within number of hours** | **Open** | **Laparoscopic** | **Robot-assisted laparoscopic** | **Open** | **Laparoscopic** | **Robot-assisted laparoscopic** |
| 0–3 | ☐ | ☐ | ☐ | ☐ | ☐ | ☐ |
| >3–6 | ☐ | ☐ | ☐ | ☐ | ☐ | ☐ |
| >6–9 | ☐ | ☐ | ☐ | ☐ | ☐ | ☐ |
| >9–12 | ☐ | ☐ | ☐ | ☐ | ☐ | ☐ |
| >12–15 | ☐ | ☐ | ☐ | ☐ | ☐ | ☐ |
| >15–18 | ☐ | ☐ | ☐ | ☐ | ☐ | ☐ |
| >18–21 | ☐ | ☐ | ☐ | ☐ | ☐ | ☐ |
| >21–24 | ☐ | ☐ | ☐ | ☐ | ☐ | ☐ |

## Thank you for your participation!

**/The SOMBATA Team**

**
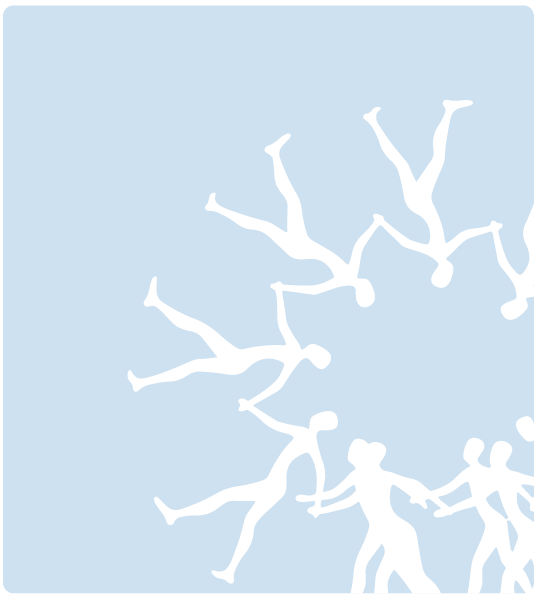
**

**FOR INFORMATION, PLEASE CONTACT:**
Monika Fagevik Olsén, Primary Investigator
Email: monika.fagevik-olsen@gu.se
